# Association between the dietary index for gut microbiota and female infertility: a cross-sectional study of NHANES 2013–2018

**DOI:** 10.3389/fnut.2025.1583805

**Published:** 2025-04-28

**Authors:** Xiaoyan Zhang, Liangzhi Wu, Haiyan Li, Shuyao Zhang, Wenfeng Hua

**Affiliations:** ^1^Department of Pharmacy, Guangzhou Red Cross Hospital (Guangzhou Red Cross Hospital of Jinan University), Guangzhou, Guangdong, China; ^2^Department of Gynecology, The Affiliated Guangdong Second Provincial General Hospital of Jinan University, Guangzhou, Guangdong, China; ^3^Department of Reproductive Medicine Center, The Affiliated Guangdong Second Provincial General Hospital of Jinan University, Guangzhou, Guangdong, China; ^4^Research Institute for Maternal and Child Health, The Affiliated Guangdong Second Provincial General Hospital of Jinan University, Guangzhou, Guangdong, China

**Keywords:** DI-GM, dysbiosis, gut microbiota, female infertility, NHANES

## Abstract

**Background:**

Infertility poses a substantial societal and economic burden; however, current preventive strategies are limited. Recently, the relationship between gut microbiota and infertility has garnered increasing attention. The dietary index for gut microbiota (DI-GM) is a new index that reflects the diversity of the gut microbiota. However, its association with female infertility remains unclear.

**Methods:**

This cross-sectional study included 3,053 women aged 18–45 years from the National Health and Nutrition Examination Survey (NHANES) database between 2013 and 2018. Infertility was defined based on responses to a questionnaire on reproductive health. The DI-GM score was calculated by averaging the intake from two 24-h dietary recall interviews. Weighted multivariable logistic regression, restricted cubic splines (RCS), and subgroup analyses were used to investigate the association between DI-GM and female infertility.

**Results:**

Based on self-reported data, 370 participants (12.12%) were classified as infertile. A higher proportion of participants with lower DI-GM scores experienced infertility. Multivariable logistic regression analysis indicated a negative association between DI-GM and the risk of female infertility, regardless of whether the independent variable was analyzed as a continuous variable or in quartiles in the fully adjusted model (Model 3, continuous variable: OR = 0.89, 95% confidence interval (CI): 0.80–0.98, *p* = 0.025; Q4 *vs.* Q1: OR = 0.63, 95% CI = 0.42–0.94, *p* = 0.032, *p* for trend = 0.013). The RCS curves demonstrated a non-linear relationship between the DI-GM scores and infertility risk. Subsequent subgroup analyses corroborated the robustness of these findings.

**Conclusion:**

These findings suggest a non-linear relationship between DI-GM and the risk of infertility in females, with lower DI-GM scores associated with a higher risk of infertility.

## Introduction

Infertility is a widespread chronic condition defined as the inability to achieve clinical pregnancy after 12 months of regular unprotected sexual intercourse. Globally, approximately one in eight couples of childbearing age experience infertility or difficulty in maintaining pregnancy (1, 2). Female infertility is influenced by genetic, environmental, and lifestyle factors. Common etiologies include ovulatory dysfunction, endometriosis, polycystic ovary syndrome (PCOS), fallopian tube abnormalities, and immunological disorders (3). Numerous studies have identified various lifestyle factors, including dietary patterns, that are correlated with infertility in women (4–6). Factors such as smoking, alcohol consumption, obesity, chronic stress, and inadequate sleep adversely affect reproductive health. Inappropriate dietary habits are often associated with either excessive or insufficient calorie intake. Deficiency in essential nutrients can delay the onset of puberty and elevate the risk of ovulation disorders, thereby reducing fertility in women (7, 8). Given that infertility has emerged as the third most significant health issue following cancer and cardiovascular diseases, it presents medical challenges and engenders social issues with substantial economic and psychosocial implications (9). There is an urgent need to develop effective strategies to prevent and manage infertility, which poses a substantial threat to public health.

The gut microbiota is essential for human health and affects various physiological processes, such as nutrient absorption, intestinal mucosal growth, glycolipid metabolism, neurological function, and immune regulation (10–12). Recent studies have indicated that an imbalance in gut microbiome composition is closely linked to female reproductive diseases such as endometriosis, chronic pelvic pain, premature ovarian failure, ovarian aging, and PCOS (13–18). Notably, Qi et al. demonstrated that patients with PCOS have elevated levels of the gut microbe *Bacteroides vulgatus* and decreased concentrations of glycodeoxycholic acid and tauroursodeoxycholic acid. Mice that received fecal microbiota transplants from patients with PCOS or those colonized by *Bacteroides vulgatus* exhibited decreased interleukin-22 secretion and increased impairment of ovarian function, insulin resistance, altered bile acid metabolism, and infertility (17). Mikkelsen et al. reported that preconception antibiotic use, especially macrolides and sulfonamides, is associated with increased infertility risk (19). These findings suggest that dysbiosis of the gut microbiota and its metabolites can increase the risk of infertility.

Accumulating evidence indicates that dietary patterns substantially influence the composition of the gut microbiota and have been implicated in the etiology of female infertility (20–24). The Mediterranean diet (MD) is characterized by the traditional dietary patterns of populations residing in countries bordering the Mediterranean Sea. This dietary regimen emphasizes the consistent consumption of fruits, vegetables, legumes, whole grains, nuts, and olive oil as fundamental components of the daily nutritional intake. Studies have shown that the MD significantly improves human metabolic health. The main reason for these benefits is the ability of the MD to regulate the gut microbiota structure and function by increasing the alpha diversity indices of the gut microbiome (25–27). Studies have demonstrated that the MD positively impacts reproductive health, with a higher percentage of clinical pregnancies and live births (28–30). In contrast, the Western diet (WD) is characterized by an abundance of calories and a scarcity of fruits, vegetables, whole grains, fish, nuts, and seeds. This eating pattern is often linked to obesity, which can alter the gut microbiota composition. These changes in the gut microbiome can significantly affect fertility in both females and males (31–34).

Dietary patterns play a significant role in shaping gut microbiota composition, making it crucial to use dietary indices to understand the relationship between the gut microbiome and disease risk (20, 35). Commonly used indices include the Healthy Eating Index (HEI), Dietary Approaches to Stop Hypertension (DASH), and Mediterranean Diet Score (MDS) (36). Although these indices have demonstrated utility in assessing the correlation between dietary quality and health outcomes, investigations into their relationships with gut microbiota diversity and abundance have produced inconsistent results (37, 38). Kase et al. developed a new dietary index for gut microbiota (DI-GM) to address this inconsistency. This index evaluates the influence of diet on the gut microbiota through 14 components identified as beneficial or unfavorable to gut health, effectively capturing the relationship between dietary quality and gut microbiota diversity (39).

Recent findings have demonstrated that a lower DI-GM score is associated with a higher risk of diabetes, stroke, constipation, metabolic dysfunction-associated fatty liver disease, depression, and aging (40–45). Diabetes, depression, and aging are closely associated with infertility in women. However, the relationship between DI-GM and infertility remains unexplored. This cross-sectional investigation aimed to address this knowledge gap by examining the association between DI-GM and female infertility using NHANES data. This study also sought to elucidate valuable information for developing targeted dietary interventions to mitigate infertility.

## Methods

### Data source

This study analyzed data from the 2013–2018 NHANES, a comprehensive cross-sectional survey conducted biennially to collect data on the dietary habits, nutritional status, health conditions, and lifestyle behaviors of the non-institutionalized U.S. population using a multistage probability sampling methodology. These data are publicly accessible through the National Center for Health Statistics (NCHS), a division of the Centers for Disease Control and Prevention (CDC). The NCHS Ethics Review Board approved the NHANES protocols, and all participants provided written informed consent. Further information can be found at http://www.cdc.gov/nchs/nhanes/index.htm.

### Study design and population

**Figure 1:**
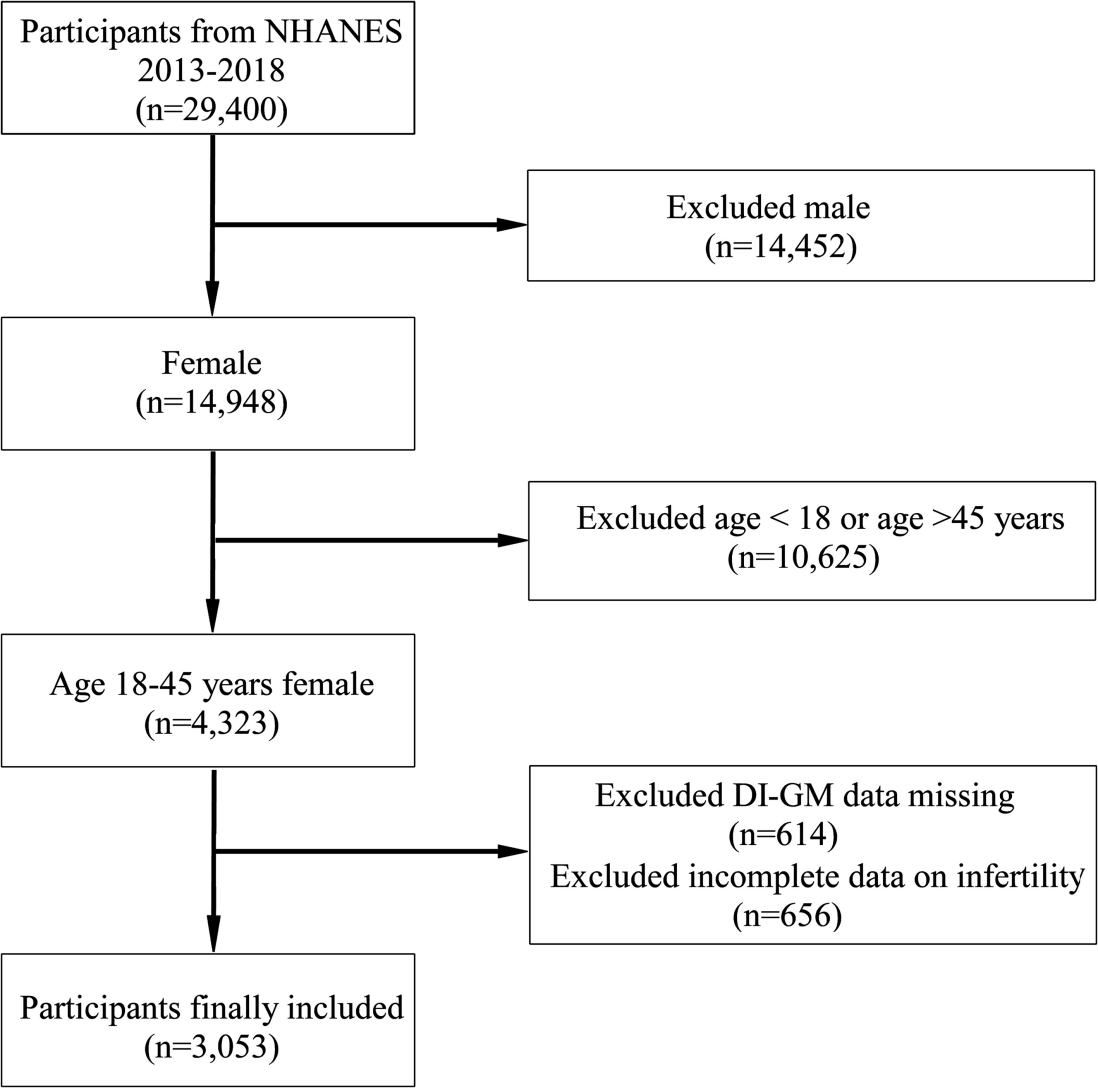
Flow chart for the inclusion and exclusion of study participants.

Our study focused on women aged 18–45 years who were not pregnant, representing the non-institutionalized civilian population in the United States. A comprehensive dataset encompassing DI-GM components and infertility status was acquired from an initial cohort of 29,400 study participants. The study excluded several groups of participants: males (*n* = 14,452), females outside the age range of 18–45 years (*n* = 10,625), individuals lacking DI-GM components (*n* = 614), and those without information on infertility (*n* = 656). The final analysis sample comprised 3,053 eligible participants (Figure 1).

### Calculation of DI-GM

This study utilized the scoring system developed by Kase et al. to calculate the DI-GM using 14 foods and nutrients (39). The DI-GM includes 10 beneficial components (avocado, broccoli, chickpeas, coffee, cranberries, fermented dairy, fiber, green tea, soy, and whole grains) and four unfavorable components (red meat, processed meat, refined grains, and high-fat diets). We averaged the results from two 24-h dietary recall interviews for each participant to calculate the DI-GM scores. Participants with only one reliable dietary recall were excluded from the analysis (39). A score of 1 indicates consumption above the median for beneficial components and below the median for detrimental components, and a score of 0 indicates the opposite. The total score ranged from 0 to 13, with higher scores suggesting a healthier gut microbiota. Based on previous studies (41–43), participants were categorized into four groups: 0–3, 4, 5, and ≥ 6.

### Definition of infertility

The dependent variable, infertility, was evaluated using two questions from the Reproductive Health Questionnaire. The first question (RHQ074) asked, “Have you ever tried unsuccessfully to conceive for a minimum of 1 year?” The second question (RHQ076) inquired, “Have you ever sought medical advice from a doctor or healthcare provider regarding difficulties in becoming pregnant?” Respondents who answered either question affirmatively were categorized as experiencing infertility, whereas those who answered both questions negatively were classified as not experiencing infertility.

### Covariates

This study considered various factors, including demographic characteristics (age, race, marital status, education level, and poverty income ratio), lifestyle habits (alcohol consumption and smoking), health conditions (hypertension, diabetes, and dyslipidemia), and reproductive health factors (age at first menstruation, history of pelvic infection or inflammatory disease, use of birth control pills, and hormone therapy). Comprehensive information on the methods used to collect data on these variables can be found on the official NHANES website.

### Statistical analysis

The surveys utilized a complex multistage clustered design, and all statistical analyses adhered to the NHANES sampling weights, as recommended by the CDC. We compared the participants’ baseline characteristics in the descriptive analyses based on infertility status and DI-GM quartiles. Continuous variables are reported as means with standard errors (SE), and categorical variables are expressed as numerical counts and percentage frequencies (%). A weighted linear regression model and chi-square tests were used to evaluate the baseline characteristics. For variables with missing data, continuous variables were imputed using the medians or means, depending on the distribution, and categorical variables were imputed using the modes.

We used weighted multivariate logistic regression models to examine the association between DI-GM and infertility risk while accounting for known or potential confounding variables. We also conducted subgroup analyses to explore the association between DI-GM and infertility across different demographic and clinical groups, considering factors such as age, BMI, PIR, PID, smoking, female hormones, and the presence of hypertension, dyslipidemia, and diabetes mellitus. We further employed restricted cubic spline (RCS) curves and threshold effect analysis to investigate the potential non-linear relationship between the DI-GM scores and infertility risk. Statistical analysis and data handling were conducted using R (version 4.4.0) and Zstats (version 1.0) software. Statistical significance was set at *p* < 0.05.

## Results

### Participant characteristics

As shown in Table 1, among the 3,053 eligible participants, 370 were infertile. Individuals in the infertile group tended to be older, have a higher body mass index (BMI), higher income, earlier age at menarche, and a higher prevalence of conditions such as being married, smoking, history of pelvic inflammatory disease (PID), use of female hormones, hypertension, dyslipidemia, and diabetes than those in the non-infertile group. Furthermore, the average DI-GM value was significantly lower in the infertility group than that in the non-infertility group (4.69 *vs.* 5.01, *p* = 0.025). Participants with lower DI-GM scores also exhibited higher triglyceride levels and fasting plasma glucose (FPG) and lower high-density lipoprotein cholesterol (HDL-C) levels (*p* < 0.05). Additionally, the prevalence of infertility among participants significantly decreased from Q1 to Q4 (*p* = 0.041, Supplementary Table S1), with notably lower rates observed in Q3 and Q4 (11.18 and 11.17%, respectively) than in Q1 and Q2 (16.32 and 16.16%, respectively). These observed variations suggest that the potential association between DI-GM and infertility requires further investigation.

**Table 1:** Basic characteristics of participants according to infertility status*.

Variable	Total (*n* = 3,053)	Non-infertility (*n* = 2,683)	Infertility (*n* = 370)	*p* value
Age, mean (SE), year	31.36 (0.26)	30.84 (0.26)	34.74 (0.56)	<0.001
BMI, mean (SE), kg/m^2^	29.27 (0.28)	28.83 (0.27)	32.17 (0.81)	<0.001
PIR, mean (SE)	2.60 (0.07)	2.56 (0.07)	2.81 (0.11)	0.040
Menarche, mean (SE), year	12.58 (0.04)	12.61 (0.04)	12.36 (0.13)	0.048
Triglyceride, mean (SE), mg/dL	88.90 (1.51)	87.25 (1.71)	99.77 (5.51)	0.048
Fasting blood glucose, mean (SE), mg/dL	97.88 (0.43)	97.52 (0.42)	100.28 (1.32)	0.045
HDL-C, mean (SE), mg/dL	57.22 (0.49)	57.68 (0.50)	54.24 (1.29)	0.013
Race, *n* (%)				0.380
Mexican American	532 (13.17)	475 (13.37)	57 (11.84)	
Other Hispanic	316 (7.25)	289 (7.51)	27 (5.57)	
Non-Hispanic White	1,007 (54.88)	858 (54.01)	149 (60.61)	
Non-Hispanic Black	689 (13.91)	612 (14.17)	77 (12.24)	
Other Race - Including Multi-Racial	509 (10.78)	449 (10.94)	60 (9.73)	
Marital status, *n* (%)				<0.001
Married	1,361 (46.55)	1,137 (44.29)	224 (61.45)	
Widowed	14 (0.56)	11 (0.30)	3 (2.30)	
Divorced	207 (6.63)	185 (6.67)	23 (6.35)	
Separated	120 (3.05)	107 (3.12)	13 (2.59)	
Never married	928 (29.92)	865 (32.08)	63 (15.61)	
Living with partner	423 (13.29)	379 (13.53)	44 (11.69)	
Education level, *n* (%)				0.152
Less than high school	513 (12.19)	461 (12.33)	52 (11.33)	
High school or equivalent	684 (21.25)	613 (21.97)	71 (16.47)	
College or above	1856 (66.56)	1,609 (65.70)	247 (72.20)	
Hypertension, *n* (%)				<0.001
Yes	435 (11.69)	351 (10.37)	84 (20.41)	
No	2,618 (88.31)	2,332 (89.63)	286 (79.59)	
Dyslipidemia, *n* (%)				0.001
Yes	387 (12.10)	315 (10.99)	72 (19.48)	
No	2,666 (87.90)	2,368 (89.01)	298 (80.52)	
Diabetes, *n* (%)				<0.001
Yes	125 (3.52)	96 (2.94)	29 (7.30)	
No	2,928 (96.48)	2,587 (97.06)	341 (92.70)	
PID, *n* (%)				<0.001
Yes	145 (4.86)	108 (3.84)	37 (11.62)	
No	2,908 (95.14)	2,575 (96.16)	333 (88.38)	
Smoking status, *n* (%)				<0.001
Yes	795 (29.27)	664 (27.78)	131 (39.13)	
No	2,258 (70.73)	2019 (72.22)	239 (60.87)	
Drinking status, *n* (%)				0.109
Yes	2,113 (74.88)	1837 (74.26)	275 (78.91)	
No	940 (25.12)	846 (25.74)	95 (21.09)	
Birth control pills, *n* (%)				0.071
Yes	1955 (71.42)	1,676 (70.62)	280 (76.65)	
No	1,098 (28.58)	1,007 (29.38)	90 (23.35)	
Female hormones, *n* (%)				0.033
Yes	197 (6.08)	167 (5.37)	30 (10.77)	
No	2,856 (93.92)	2,516 (94.63)	340 (89.23)	
DI-GM MEAN, mean (SE)	4.97 (0.05)	5.01 (0.05)	4.69 (0.13)	0.025

BMI, body mass index; HDL-C, high-density lipoprotein cholesterol; PIR, poverty impact ratio; PID, pelvic infection/inflammatory disease; DI-GM, dietary index for gut microbiota.

^*^Percentage estimates are nationally representative using survey weights.

### Association between DI-GM and infertility

The correlation between the DI-GM score and the risk of female infertility is shown in Table 2. Logistic regression analysis revealed a significant negative association between DI-GM scores and infertility risk. When DI-GM was used as a continuous variable, the odds ratio (OR) of Model 1 (M1) was 0.89 (95% CI = 0.81–0.99, *p* = 0.029). After adjusting for demographic factors (M2) and in the fully adjusted model (M3), the OR values remained significant (M2: OR = 0.87, 95% CI = 0.79–0.96, *p* = 0.010; M3: OR = 0.89, 95% CI = 0.80–0.98, *p* = 0.025). Further analysis based on the DI-GM score groupings supported these findings. The results indicated that the highest group (Q4) was significantly associated with a reduced risk of infertility compared with the lowest group (Q1) across all three models (M1: OR = 0.65, 95% CI = 0.45–0.93, *p* = 0.022; M2: OR = 0.58, 95% CI = 0.39–0.86, *p* = 0.010; and M3: OR = 0.63, 95% CI = 0.42–0.94, *p* = 0.032). Moreover, trend analyses across all models demonstrated statistical significance (*p* < 0.05), further supporting the strong association between higher DI-GM scores and a decreased risk of infertility.

**Table 2:** Association between DI-GM and female infertility.

Variables	Model 1		Model 2		Model 3	
	OR (95%CI)	*p* value	OR (95%CI)	*p* value	OR (95%CI)	*p* value
DI-GM	0.89 (0.81–0.99)	0.029	0.87 (0.79–0.96)	0.010	0.89 (0.80–0.98)	0.025
DI-GM group						
Q1 (0–3)	1.00 (Reference)		1.00 (Reference)		1.00 (Reference)	
Q2 (4)	0.99 (0.72–1.36)	0.944	0.99 (0.69–1.41)	0.947	1.05 (0.73–1.50)	0.812
Q3 (5)	0.65 (0.39–1.06)	0.090	0.60 (0.37–0.97)	0.044	0.61 (0.38–0.98)	0.050
Q4 (≥6)	0.65 (0.45–0.93)	0.022	0.58 (0.39–0.86)	0.010	0.63 (0.42–0.94)	0.032
*p* for trend	0.012		0.004		0.013	

Model 1: Crude.

Model 2: Adjusted for age, ethnicity, education level, PIR, and marital status.

Model 3: Further adjusted for smoking, drinking, dyslipidemia, diabetes, hypertension, menstrual status, PID, birth control pills, and female hormones.

### Non-linear relationship between DI-GM and infertility

**Figure 2:**
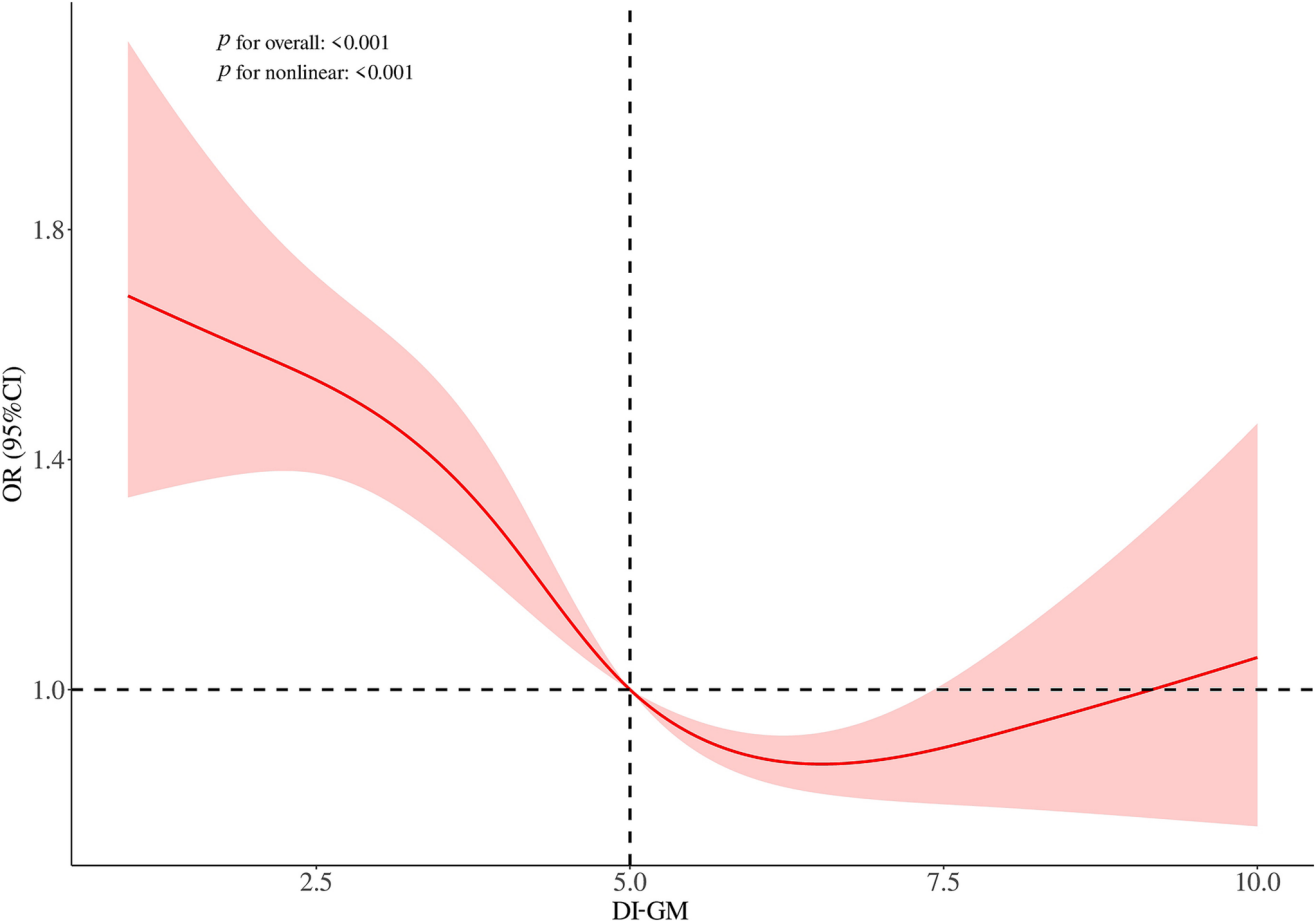
Restricted cubic spline plots for the association between DI-GM and infertility in women. Adjusted for age, ethnicity, education level, PIR, marital status, smoking, drinking, dyslipidemia, diabetes, hypertension, menstrual status, PID, birth control pill use, and female hormone use.

RCS analysis was conducted to explore the relationship between DI-GM scores and infertility risk. The findings indicated a non-linear relationship between DI-GM and infertility risk (Figure 2). To examine this relationship in greater detail, a weighted two-segment linear regression model and a recursive algorithm were employed to conduct a threshold effect analysis. The analysis identified an inflection point at a DI-GM score of 8, with a log-likelihood ratio test showing significance at *p* < 0.001. For DI-GM scores below the threshold, each unit increase in the DI-GM was linked to a 14% reduction in the risk of infertility (OR = 0.86, 95%CI = 0.83–0.90, *p* < 0.001; Table 3). Conversely, when the DI-GM scores surpassed this threshold, each incremental unit was associated with a 197% increased probability of infertility (OR = 2.97, 95% CI = 2.15–4.12, *p* < 0.001; Table 3).

**Table 3:** Threshold effect analysis of DI-GM and female infertility*.

Outcome	Effect OR (95% CI)	*p* value
DI-GM		
Model 1	0.89 (0.86–0.91)	<0.001
Model 2		
Inflection point	8	
<8	0.86 (0.83–0.90)	<0.001
≥8	2.97 (2.15–4.12)	<0.001
*p* for likelihood test		<0.001

Model 1: Fitting model using standard linear regression.

Model 2: Fitting model using two-piecewise linear regression.

^*^Adjusted for age, ethnicity, education level, PIR, marital status, smoking, drinking, dyslipidemia, diabetes, hypertension, menstrual status, PID, birth control pills, and female hormones.

### Subgroup analysis

**Figure 3:**
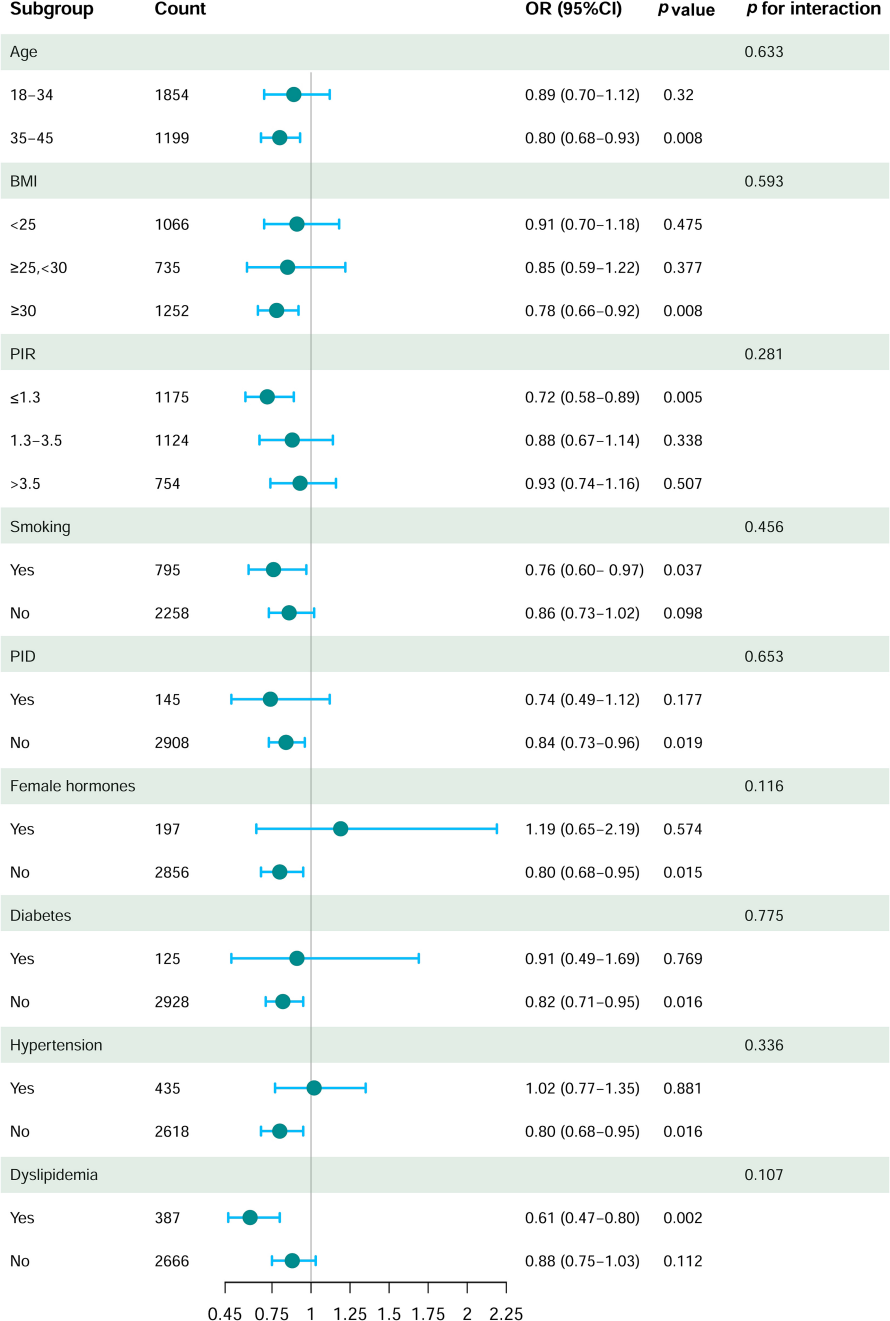
Forest plot of stratified analysis and interaction effects on the association between DI-GM and infertility in women. The model was adjusted for age, ethnicity, education level, PIR, marital status, smoking, drinking, dyslipidemia, diabetes, hypertension, menstrual status, PID, birth control pill use, and female hormone use.

To further investigate the association between DI-GM scores and the risk of female infertility, we analyzed various subgroups based on demographic and health factors. The results showed a significant inverse relationship between DI-GM and the risk of infertility, which remained consistent across various subgroups. These subgroups included individuals aged 35–45 years, those with obesity (BMI ≥ 30), individuals with lower income (PIR ≤ 1.3), smokers, those without a history of pelvic inflammatory disease (PID), and those not using female hormones. This association was also observed in individuals without hypertension or diabetes and those with dyslipidemia (*p* < 0.05, Figure 3). Furthermore, we found no statistically significant interactions between the DI-GM scores and covariates (*p* > 0.05, Figure 3). These extensive subgroup analyses provide strong evidence of a consistent association between DI-GM scores and the risk of female infertility across diverse population segments.

## Discussion

In the present study, we demonstrated a significant association between DI-GM and female infertility. Our findings showed that the non-infertile group had significantly higher DI-GM scores than the infertile group. Higher DI-GM scores were significantly inversely associated with the risk of female infertility. We found that DI-GM was protective in subgroups aged 35–45 years, with BMI ≥ 30, PIR ≤ 1.3, smokers, without PID history, and not using female hormones. Using RCS analysis, we identified a non-linear relationship between DI-GM scores and infertility risk. Furthermore, we found a significant negative association between DI-GM and female infertility risk for scores below the inflection point of eight. However, above this threshold, a positive association with infertility risk was observed.

Recent studies suggest that dysbiosis of the gut microbiota may be a potential pathogenic factor in the development of PCOS, a prevalent endocrine disorder linked to a heightened risk of infertility (17, 46, 47). Qi et al. found a notable increase in *Bacteroides vulgatus* in the gut microbiota of individuals with PCOS, leading to a decrease in interleukin-22 (IL-22) secretion in the serum and follicular fluid, which plays a crucial role in mitigating the PCOS phenotype. Wu et al. identified enriched gut *Aspergillus tubingensis* in patients with PCOS, and this fungus induced a PCOS-like phenotype by inhibiting IL-22 secretion from intestinal group 3 innate lymphoid cells (ILC3s) via inhibition of the Aryl hydrocarbon receptor (AhR)-interleukin (IL)-22 pathway in mice. Furthermore, the gut microbiota influences reproductive health through various mechanisms, including the regulation of circulating sex hormone levels, immune system function, insulin sensitivity, and interactions with the gonadal microbiota. Additionally, healthy gut microbiota may contribute to a decreased risk of female infertility by modulating estrogen metabolism and controlling systemic inflammation (13, 14, 48, 49).

Evidence suggests that dietary practices substantially influence the composition of the gut microbiota, highlighting the critical role of dietary indices in elucidating the relationship between the gut microbiome and disease risk (20, 35). A healthy gut microbiome is characterized by high richness and diversity of microorganisms. The gut microbiota influences the host response to diet, while the host can also modify the gut microbiota through changes in dietary habits. An unhealthy diet high in fat and sugar may lead to decreased microbial diversity, reduced production of metabolites that support gut permeability, damage to the mucus layer, increased bacterial translocation, and higher lipopolysaccharide levels. These changes can trigger endotoxemia, chronic subclinical inflammation, and metabolic disorders (50, 51). The health of the intestinal microbiota is significantly associated with female infertility (13, 14, 17). Therefore, evaluating gut microbiota health by assessing dietary habits among women of reproductive age is valuable for public health (4). The DI-GM is a new dietary pattern index designed to predict gut microbiota health by identifying 14 dietary components that can have beneficial or unfavorable effects on the gut microbiome (39). This study examined the relationship between DI-GM and female infertility. We found that DI-GM has a non-linear inverse associated with the risk of female infertility. Our findings suggest that elevated DI-GM scores may protect against female infertility. Additionally, we observed that individuals with higher DI-GM scores had lower triglyceride and FPG levels and higher HDL-C levels. This may be linked to insulin resistance (IR), a common cause of infertility in PCOS, suggesting that a higher DI-GM may be associated with lower IR (52).

The association between higher DI-GM scores and a lower risk of female infertility highlights the potential of dietary interventions to improve gastrointestinal health. However, we observed a positive association between DI-GM and female infertility risk when the DI-GM scores were greater than eight in the threshold effect analysis. Based on the DI-GM calculation method, as the score increases, the consumption of dietary components with high energy densities, such as red meat and high-fat milk, declines (39). Our results suggest that inadequate energy intake may occur in women of childbearing age when the DI-GM scores exceed eight. These findings indicate that the best approach for women of reproductive age is to balance beneficial ingredients for gut health, represented by DI-GM, with less favorable ingredients. A balanced and healthy diet can boost fertility and improve the chances of conception by enhancing nutritional status. This balance is essential for reproduction as it helps regulate energy and nutrition. Additionally, the interactions between diet and microbiota, which influence human metabolism, should align with the physiological needs related to reproduction (20, 53–55).

This study had several strengths. After controlling for confounding factors, this is the first study to demonstrate a significant association between DI-GM and female infertility. These results indicate that lower DI-GM scores are associated with an increased risk of infertility in females. Additionally, this study established a non-linear relationship between DI-GM and infertility risk. Subgroup analysis further reinforced the robustness of these findings. Future longitudinal studies should explore the combined effects of dietary and gut microbiota interventions on reproductive health in diverse populations to validate these findings.

Our study had several limitations. First, the cross-sectional study design restricts the ability to establish a causal relationship between DI-GM and female infertility, underscoring the need for future longitudinal and prospective studies. Second, the DI-GM scores were calculated based on the intake data from 14 food components, leading to participant exclusion when data were missing, which may have introduced a selection bias. Moreover, the dependence on self-reported dietary information and the assessment of infertility using a reproductive health questionnaire increased the potential for recall and social desirability bias in this study. Third, despite adjustments for numerous potential confounders, the possibility of residual confounding and unmeasured factors, such as dietary supplement use and undiagnosed reproductive disorders, cannot be entirely excluded. Fourth, DI-GM reflects dietary habits during data collection rather than long-term patterns; however, most adults maintain consistent diets unless they experience significant health issues, suggesting that the DI-GM reasonably represents typical dietary habits. Fifth, female infertility is affected by lifestyle factors such as occupational stress and physical activity. However, the DI-GM score does not comprehensively capture the influence of these factors on female infertility risk. Finally, the generalizability of the study findings is constrained because significant associations were primarily observed in the U.S. population. A more diverse representation of populations is necessary to validate these findings and enhance our understanding of the relationship between diet, gut microbiota, and the risk of female infertility. To determine the causal relationship between DI-GM and female infertility, future research should consider longitudinal study designs or microbiome sequencing data.

## Conclusion

This study found a significant negative association between DI-GM and the risk of infertility in women. Interestingly, the relationship between the DI-GM scores and infertility risk demonstrated a non-linear pattern. As a new dietary quality index that reflects gut microbiota diversity, further research and interventions using DI-GM could help develop strategies to prevent and reduce the risk of female infertility.

## Data Availability

The datasets presented in this study can be found in online repositories. The names of the repository/repositories and accession number(s) can be found at: https://wwwn.cdc.gov/nchs/nhanes/Default.aspx.
