# Supplementary material for: Association between the dietary index for gut microbiota and female infertility: a cross-sectional study of NHANES 2013–2018
Source: Front Nutr. 2025 Apr 28;12:1583805. doi: 10.3389/fnut.2025.1583805 (PMC12066596; doi:10.3389/fnut.2025.1583805)
Supplement: Supplementary file 1 [file Table_1.docx]

**Table S1. Basic characteristics of the study population based on** **DI-GM scores*.**

| **Variable** | **Q1**  **(0-3, n=621)** | **Q2**  **(4, n=666)** | **Q3**  **(5, n=736)** | **Q4**  **(≥6, n=1030)** | ***p* value** |
| --- | --- | --- | --- | --- | --- |
|  |  |  |  |  |  |
| **Age, mean (SE), year** | 30.97 (0.43) | 30.82 (0.41) | 31.37 (0.50) | 31.87 (0.38) | 0.069 |
| **BMI, mean (SE), kg/m^2^** | 30.29 (0.45) | 30.43 (0.54) | 28.83 (0.44) | 28.38 (0.42) | <0.001 |
| **PIR, mean (SE)** | 2.33 (0.10) | 2.23 (0.11) | 2.66 (0.11) | 2.91 (0.10) | <0.001 |
| **Menarche, mean (SE), year** | 12.52 (0.10) | 12.48 (0.11) | 12.62 (0.07) | 12.63 (0.08) | 0.285 |
| **Triglyceride, mean (SE), mg/dL** | 94.63 (4.50) | 91.23 (2.55) | 87.83 (3.64) | 85.26 (2.13) | 0.050 |
| **Fasting blood glucose,**  **mean (SE), mg/dL** | 100.38 (1.22) | 99.43 (1.17) | 96.06 (0.73) | 96.97 (0.74) | 0.004 |
| **HDL-C, mean (SE), mg/dL** | 54.89 (1.03) | 55.04 (0.82) | 58.61 (0.74) | 58.75 (0.70) | <0.001 |
| **Race, n (%)** |  |  |  |  | 0.012 |
| Mexican American | 95 (11.60) | 121 (14.53) | 144 (14.52) | 172 (12.23) |  |
| Other Hispanic | 74 (7.34) | 75 (8.40) | 62 (6.21) | 105 (7.29) |  |
| Non-Hispanic White | 186 (51.03) | 220(53.22) | 249 (55.19) | 352 (57.68) |  |
| Non-Hispanic Black | 179 (19.81) | 166 (14.98) | 161 (13.09) | 183 (10.76) |  |
| Other Race - Including  Multi-Racial | 87 (10.22) | 84 (8.88) | 120 (10.99) | 218 (12.05) |  |
| **Marital status, n (%)** |  |  |  |  | 0.153 |
| Married | 244 (44.04) | 280 (45.85) | 337 (48.54) | 500 (46.85) |  |
| Widowed | 4 (0.49) | 4 (0.56) | 4 (1.25) | 2 (0.10) |  |
| Divorced | 37 (5.90) | 56 (7.86) | 47 (5.73) | 66 (6.94) |  |
| Separated | 24 (1.80) | 29 (3.65) | 27(2.71) | 40 (3.61) |  |
| Never married | 208 (30.35) | 200 (28.33) | 227 (31.39) | 293 (29.56) |  |
| Living with partner | 104 (17.43) | 97 (13.74) | 94 (10.37) | 129(12.93) |  |
| **Education level, n (%)** |  |  |  |  | <0.001 |
| Less than high school | 113 (14.40) | 142 (14.24) | 133 (13.23) | 125 (9.09) |  |
| High school or equivalent | 161 (25.91) | 167 (26.17) | 183 (22.73) | 173 (14.84) |  |
| College or above | 347 (59.69) | 357 (59.59) | 420 (64.04) | 732 (76.08) |  |
| **Hypertension, n (%)** |  |  |  |  | 0.092 |
| Yes | 101 (16.24) | 98 (11.41) | 111 (11.17) | 125 (9.83) |  |
| No | 520 (83.76) | 568 (88.59) | 625 (88.83) | 905 (90.17) |  |
| **Dyslipidemia, n (%)** |  |  |  |  | 0.585 |
| Yes | 71 (11.38) | 74 (12.06) | 103 (10.89) | 139 (13.38) |  |
| No | 550 (88.62) | 592 (87.94) | 633 (89.11) | 891 (86.62) |  |
| **Diabetes, n (%)** |  |  |  |  | 0.001 |
| Yes | 32 (6.60) | 34 (5.22) | 26 (1.86) | 33 (2.07) |  |
| No | 589 (93.40) | 632 (94.78) | 710 (98.14) | 997 (97.93) |  |
| **PID, n (%)** |  |  |  |  | 0.510 |
| Yes | 37 (5.99) | 30 (4.67) | 34 (5.59) | 44 (3.85) |  |
| No | 584 (94.01) | 636 (95.33) | 702 (94.41) | 986 (96.15) |  |
| **Smoking status, n (%)** |  |  |  |  | 0.194 |
| Yes | 173 (32.65) | 178 (30.43) | 202 (30.53) | 242 (25.90) |  |
| No | 448 (67.35) | 488 (69.57) | 534 (69.47) | 788 (74.10) |  |
| **Drinking status, n (%)** |  |  |  |  | 0.479 |
| Yes | 431 (74.73) | 439 (72.26) | 513 (75.79) | 729 (75.83) |  |
| No | 190 (25.27) | 227 (27.74) | 223 (24.21) | 301 (24.17) |  |
| **Birth control pills, n (%)** |  |  |  |  | 0.498 |
| Yes | 377 (67.68) | 425 (71.27) | 484 (72.32) | 670 (72.84) |  |
| No | 244 (32.32) | 241 (28.73) | 252 (27.68) | 360 (27.16) |  |
| **Female hormones, n (%)** |  |  |  |  | 0.873 |
| Yes | 39 (6.88) | 47 (5.53) | 49 (6.69) | 61 (5.55) |  |
| No | 582 (93.12) | 619 (94.47) | 687 (93.31) | 969 (94.45) |  |
| **Infertility, n (%)** |  |  |  |  | 0.041 |
| Yes | 94 (16.32) | 81 (16.16) | 87 (11.18) | 108 (11.17) |  |
| No | 527 (83.68) | 585 (83.84) | 649 (88.82) | 922 (88.83) |  |

BMI, body mass index; HDL-C, high-density lipoprotein cholesterol; PIR, poverty impact ratio; PID, pelvic infection/inflammatory disease; DI-GM, dietary index for gut microbiota.

*Percentage estimates are nationally representative using survey weights.
